# M2 Macrophage Polarization Characterizes an Immunosuppressive Microenvironment in Extracranial Arteriovenous Malformations

**DOI:** 10.3390/biomedicines14071519

**Published:** 2026-07-07

**Authors:** Syed J. Mehdi, Michael A. Bauer, Haihong Zhang, Ravi W. Sun, Jordan Bowen, Stetson Van Matre, Gresham T. Richter, Graham M. Strub

**Affiliations:** 1 Arkansas Children’s Research Institute (ACRI), Little Rock, AR 72202, USA; sjmehdi@uams.edu (S.J.M.);; 2 Department of Otolaryngology-Head and Neck Surgery, College of Medicine, University of Arkansas for Medical Sciences, Little Rock, AR 72202, USA; 3 Department of Biomedical Informatics, University of Arkansas for Medical Sciences, Little Rock, AR 72205, USA

**Keywords:** extracranial arteriovenous malformation, macrophages, immunosuppression, endothelial cells, CD31, vascular anomaly

## Abstract

**Background**: Extracranial arteriovenous malformations (eAVMs) are aggressive vascular anomalies consisting of abnormal blood vessels (BVs) and multiple other cell types, including macrophages. Although inflammation and the presence of immune cells are characteristics of eAVMs, the contribution of macrophage polarization to eAVM pathophysiology is unknown. **Methods**: In this study, pediatric eAVM tissues and adjacent control tissues were analyzed using immunohistochemistry (IHC) and immunofluorescence (IF) to assess M1 and M2 macrophage localization, loss of endothelial CD31 expression, and expression of the immune-regulatory protein PDL-1. In addition, serum samples from eAVM patients were analyzed using a human inflammation antibody array to profile cytokines and other circulating factors associated with M2 macrophage and immunosuppressive microenvironment. **Results**: eAVM tissues demonstrate accumulation of M2-polarized macrophages around abnormal CD31^−^^ve^ BVs, while M1 macrophages were primarily associated with normal appearing CD31^+ve^ vessels. eAVM tissues demonstrated increased expression of PD-L1 in regions enriched with M2 macrophages, which were absent in paired control tissues. Serum analysis revealed increased levels of circulating factors associated with M2 macrophages and immune suppression, including PDGF-BB, IL-4, and IL-16. **Conclusions**: These findings suggest that CD31^−ve^ vessels in eAVMs are associated with enrichment of M2 macrophages and a microenvironment suggestive of localized immune regulation. These observations are hypothesis-generating and warrant validation in larger patient cohorts and future mechanistic studies.

## 1. Introduction

Extracranial arteriovenous malformations (eAVMs) are congenital vascular anomalies characterized by abnormal direct connections between arteries and veins that bypass the capillary network [1]. eAVMs are rare, affecting fewer than 1 in 100,000 individuals, and their underlying pathogenesis remains poorly understood [2]. eAVMs most commonly affect the head and neck region, but may involve any part of the body [3]. Clinically, these lesions are aggressive and can rapidly expand, hemorrhage, cause pain, and result in significant disfigurement, with disease progression often accelerating during adolescence and substantially affecting physical and mental health [4,5]. These lesions are highly angiogenic and locally invasive, and no current therapy reliably achieves long-term disease control [6,7]. Management is largely palliative, as surgical and medical interventions may reduce symptoms and burden of disease, but rarely prevent recurrence [8].

Histologically, eAVMs are distinct from normal tissue due to excessive angiogenesis and pronounced perivascular tissue remodeling [9]. Recent genetic studies have identified somatic single-nucleotide variants (SNVs) in genes involved in the RAS/MAPK signaling pathway, including RASA1, MAP2K1, KRAS, and BRAF, in eAVMs [10,11,12]. These mutations are believed to disrupt normal endothelial cell (EC) signaling and contribute to abnormal vascular growth and remodeling. However, the biological mechanisms that connect these genetic alterations to lesion progression remain incompletely understood. One potential mechanism is endothelial-to-mesenchymal transition (EndMT), a process in which ECs lose their normal vascular identity and acquire mesenchymal characteristics that promote vascular remodeling and inflammation [13]. EndMT may influence the vascular microenvironment by altering cytokine production and recruiting inflammatory cells, including macrophages [14]. In turn, macrophages, particularly M2-polarized macrophages, can further promote vascular remodeling and angiogenesis through the secretion of growth factors and cytokines [15,16]. These observations suggest that genetic mutations affecting endothelial signaling may contribute to disease progression not only through direct vascular effects but also by shaping the immune microenvironment. Understanding how these genetic drivers interact with EndMT and macrophage polarization may therefore provide important insight into the mechanisms underlying eAVM progression and may identify new therapeutic targets.

Increasing evidence also suggests that the local microenvironment, including abnormal interactions among ECs, mural cells (MCs), and immune cells, plays a critical role in driving the unstable and rapidly expanding vasculature characteristic of eAVMs [17,18]. MCs regulate angiogenesis, vascular stabilization, and permeability [19,20], interacting with neighboring ECs through both physical contact and paracrine signaling [21,22]. Macrophages additionally influence vascular remodeling through secretion of growth factors and cytokines that affect EC behavior [23,24,25,26].

Emerging evidence highlights the critical role of inflammation in the progression and rupture of brain arteriovenous malformations (bAVMs) [27,28,29,30], which leads to vascular instability and increased rupture risk [31,32]. Macrophages and other inflammatory cells are often observed in the vascular walls and the underlying stroma of bAVMs, even in lesions without a history of rupture [28,33,34]. Ruptured bAVMs also exhibit a high density of M2-polarized macrophages [25,27], resembling tumor-associated macrophages (TAMs) or M2 macrophages in malignancies, which promote angiogenesis, growth, and metastasis [35,36,37]. Previous studies suggest that macrophages are a dominant inflammatory cell population in bAVMs and may correlate with lesion severity [27]. This aligns with studies showing increased bAVM macrophage burden in adult heterozygous null Eng or Alk1 HHT mice (Eng−/+, Alk1−/+) subjected to focal angiogenic stimulation [38,39,40,41]. Although macrophage infiltration contributes to bAVM pathophysiology [25,27,28], the mechanisms involved and their role in eAVMs remain poorly understood.

We recently described a population of abnormally shaped BVs lined by CD31^−ve^ vascular endothelial cells in eAVMs and demonstrated that these vessels undergo EndMT [18]. Because EndMT has been associated with altered immune signaling and M2 macrophage polarization in other disease contexts [42,43,44,45], we sought in this study to determine whether these EndMT-rich regions of eAVMs also demonstrate M2 macrophage polarization. We also sought to determine whether M2 macrophage polarization is associated with a locally immunoregulatory microenvironment by analyzing PD-L1 expression in eAVM tissues compared to normal controls. Finally, we analyzed whether known circulating biomarkers of immunosuppression were elevated in the serum of eAVM patients compared to age-matched controls.

## 2. Materials and Methods

### 2.1. Study Approval

The study was conducted under an ongoing Institutional Review Board (IRB) approved biospecimen repository protocol (IRB #114012), which undergoes annual continuing review and approval. Human tissue and serum samples used in this study were obtained from specimens collected under this IRB-approved repository protocol. Written informed consent was obtained from the parents or legally authorized representatives of all pediatric participants, and assent was obtained from children 7–17 years of age when appropriate, in accordance with the IRB-approved consent process.

### 2.2. Primary Human Samples

eAVM tissues and normal tissue biopsies (taken from nearby, unaffected subcutaneous tissue) were collected from pediatric patients during surgical resections and were deidentified. All samples came from the head and neck area. After the tissue was removed, part of each specimen was placed in 10% neutral-buffered formalin for histology. Blood samples were centrifuged, the serum was divided into aliquots, and the samples were immediately stored at −80 °C for later cytokine analysis. The tissue and serum analyses were performed using independent patient cohorts collected under the same IRB-approved study protocol.

### 2.3. Immunohistochemistry

Immunohistochemistry (IHC) was performed on paraffin-embedded sections as previously described [46,47]. Heat-mediated antigen retrieval was carried out in Tris/EDTA buffer (pH 9.0) for 30 min, followed by blocking with normal serum for 20 min. Slides were then incubated with the primary antibody for 30 min, with 5-min washes between steps. Antibodies used are listed in Table S1. Staining was developed with the Vectastain Elite ABC peroxidase (HRP) kit (Vector Laboratories, Burlington, ON, Canada), and sections were counterstained with hematoxylin. Images were taken on an Olympus BX43 microscope (Olympus, Tokyo, Japan) at 20× and 40× magnification using an Infinity 3S digital camera (Teledyne Lumenera, Ottawa, ON, Canada) in bright-field mode. Images were captured with Infinity Analyze 7 software and processed to 600 dpi using Photoshop CS6 (Adobe, San Jose, CA, USA).

### 2.4. Immunofluorescence Staining

Immunofluorescence staining was performed on paraffin-embedded sections as previously described [18,48]. Briefly, slides were deparaffinized in xylene and followed by rehydration through a graded ethanol series. Heat-mediated antigen retrieval was performed in Tris/EDTA buffer (pH 9.0) for 30 min, followed by blocking with normal serum for 20 min. Slides were then incubated with primary antibodies at optimized concentrations for 2 h at room temperature. Slides were washed with PBS and incubated in the dark for 2 h with a mixture of 2 fluorescent secondary antibodies: Alexa Fluor 488 goat anti-rabbit IgG (Invitrogen/Thermo Scientific, Waltham, MA, USA) for detecting CD204 and Alexa Flour 594 goat anti-mouse IgG (Invitrogen/Thermo Scientific, Waltham, MA, USA) for detecting CD163. Nuclei were counterstained with DAPI (1:1000) for 5 min, followed by PBS wash. Finally, slides were mounted with ProLong Gold antifade reagent (Thermo Scientific, Waltham, MA, USA) and coverslipped. Images were taken using Infinity Analyze v7.1 software (Teledyne Lumenera, Ottawa, ON, Canada) and further processed to 600 dpi using Photoshop CS6 (64 bit) (Adobe, San Jose, CA, USA). For CD31/CD163 double immunofluorescence staining, the same staining procedure was performed using mouse anti-CD31 and rabbit anti-CD163 primary antibodies. FITC-conjugated goat anti-mouse IgG was used to detect CD31, and TRITC-conjugated goat anti-rabbit IgG was used to detect CD163. Nuclei were counterstained with DAPI (1:1000) for 5 min, and slides were mounted and imaged as described above. Antibodies used are listed in Table S1.

### 2.5. Human Inflammation Antibody Array

To simultaneously quantify 40 inflammation-associated proteins in serum, a membrane-based human inflammation antibody C3 array (C-Series, AAH-INF-3; RayBiotech, Norcross, GA, USA) was used. Serum samples from four age-matched healthy controls and five patients with eAVM were processed according to the manufacturer’s instructions. Briefly, 1 mL of each diluted serum sample was applied to a pre-blocked membrane and incubated overnight at 4 °C with gentle agitation. Membranes were subsequently incubated with a biotin-conjugated primary antibody for 2 h, followed by incubation with horseradish peroxidase (HRP)-conjugated streptavidin for 2 h at room temperature. Signal intensities were visualized using the ImageQuant™ LAS 4000 detection system (GE Healthcare, Chicago, IL, USA), and spot densities were quantified with ImageJ software (Version 1.54r, National Institutes of Health, Bethesda, MD, USA). Raw spot intensity values were normalized to the internal positive control spots present on each membrane according to the manufacturer’s recommendations to reduce inter-membrane variability. The resulting densitometry values were imported into Microsoft Excel for downstream statistical analysis and visualization. A heatmap was generated in R using the pheatmap package (version 1.0.13). Expression values for genes were scaled to z-scores across samples prior to visualization. Rows (genes) were hierarchically clustered using Canberra distance and Ward.D2 linkage, while columns (samples) were displayed in their original order and annotated by condition (Normal vs. eAVMs).

### 2.6. Statistical Analysis

All values are expressed as mean ± standard error of the mean unless otherwise indicated. Differences were considered statistically significant at *p* < 0.05. Serum antibody array data were analyzed as an exploratory dataset intended to identify candidate immune-associated proteins rather than validated biomarkers. An unpaired two-tailed Student’s *t*-test was selected because comparisons were performed between two independent groups. Given the exploratory nature of the serum antibody array and the limited sample size, the statistical analyses were intended to identify candidate immune-associated proteins for future validation rather than establish definitive biomarkers. Formal multiple-comparison correction was not applied because of the limited sample size and exploratory nature of the study. Mean protein density values are reported with corresponding *p*-values where applicable.

## 3. Results

### 3.1. M2 Macrophage Accumulation in CD31^−ve^ eAVM BVs

**Figure 1:**
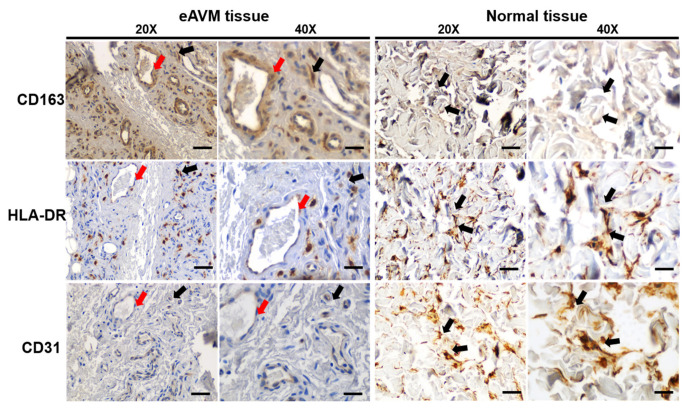
M2 macrophages are enriched around abnormal vessels in eAVM tissue. Sequential paraffin embedded sections from eAVM lesion tissue and paired adjacent unaffected control tissue from three patients were stained for CD163 (M2 macrophage), HLA-DR (M1 macrophage), and CD31 (mature EC marker). Normal-caliber CD31^+ve^ vessels are indicated by black arrows, and dilated CD31^−ve^ vessels by red arrows. Representative immunohistochemical images demonstrate that CD163^+ve^ M2 macrophages preferentially localize around CD31^−ve^ abnormal vessels, whereas HLA-DR^+ve^ M1 macrophages are associated with normal CD31^+ve^ vessels and normal tissue. Magnification = 20x and 40x; Bar = 200 μm and 100 μm.

Because accumulation of M2 macrophages is a known driver of pathogenesis in cancer and other vascular diseases [49,50,51,52,53,54], we compared macrophage polarization in eAVM tissues and paired normal control tissues by analyzing CD163^+ve^ (M2) and HLA-DR^+ve^ (M1) expression. Overall, CD163 immunoreactivity appeared increased in eAVM tissues compared to controls, indicating a high degree of M2 polarization in eAVMs (Figure 1 and Figure S1). Interestingly, CD163 expression was localized around dilated, abnormally shaped BVs within the eAVM. In contrast, HLA-DR^+ve^ M1 macrophages were detected around all CD31^+ve^ BVs, both within the eAVM and in adjacent control tissues.

**Figure 2:**
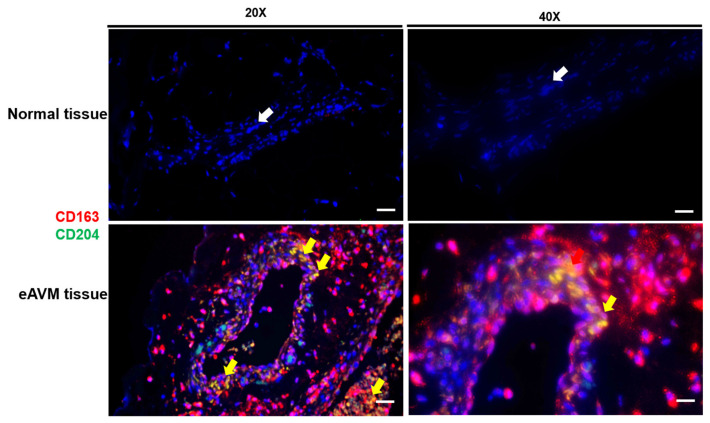
Co-localization of M2 macrophage markers around abnormal eAVM vessels. Paraffin embedded sections from eAVM lesion tissue and paired adjacent unaffected control tissue from three patients were stained for M2 macrophage markers: CD163 and CD204. Representative immunofluorescence staining confirms co-localization of M2 macrophages around abnormal eAVM vessels, with little to no expression in normal tissue vessels. Normal vessels are indicated by white arrows, and abnormal vessels by yellow arrows. Magnification = 20x and 40x; Bar = 200 μm and 100 μm.

To demonstrate whether this accumulation of M2 macrophages was occurring at BVs that had lost CD31 during EndMT, as we have previously observed [18], we validated our findings by co-staining for CD31, CD163, and an additional M2 macrophage marker, CD204. Co-staining for CD163 and CD204 confirmed M2 macrophage polarization surrounding abnormally dilated vessels, whereas normal vessels showed no detectable expression of either marker (Figure 2).

**Figure 3:**
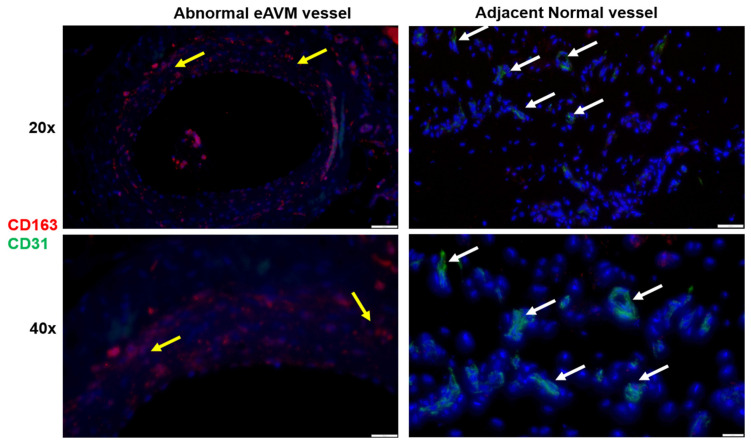
CD163^+ve^ macrophages localize around CD31^−ve^ abnormal vessels in eAVM tissue. Representative immunofluorescence images of paraffin-embedded eAVM lesion tissue and paired adjacent unaffected control tissue sections from three patients show expression of the M2 macrophage marker CD163 localized around abnormal CD31^−ve^ eAVM vessels, while CD163^+ve^ cells are absent in regions containing adjacent CD31^+ve^ vessels of normal caliber. Yellow arrows indicate ECs within regions containing dilated abnormal CD31^−ve^ vessels, whereas white arrows indicate ECs in adjacent regions of the same specimen with a high density of normal-caliber CD31^+ve^ vessels. Magnification = 20x and 40x; Bar = 200 μm and 100 μm.

In addition, co-staining for CD31 and CD163 demonstrated that CD163^+ve^ macrophages were localized around CD31^−ve^ abnormal vessels, whereas CD163^+ve^ cells were absent around adjacent CD31^+ve^ vessels of normal caliber (Figure 3 and Figure S2). Together, these results demonstrate that abnormal CD31^−ve^ vessels in eAVMs are selectively infiltrated by M2 macrophages, while M1 macrophages are primarily associated with normal tissue and normal-appearing CD31^+ve^ vessels in both control tissues and within the eAVM itself.

### 3.2. PD-L1 Expression Is Increased in M2-Enriched eAVM Regions

**Figure 4:**
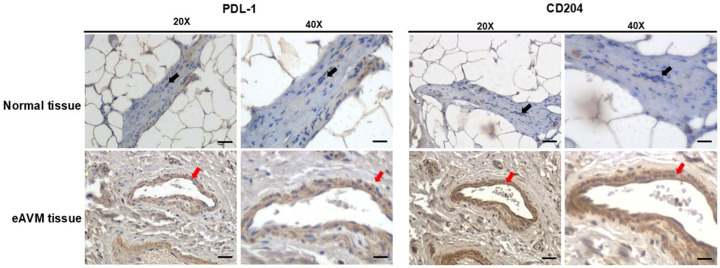
Increased PD-L1 expression in regions enriched for M2 macrophages in eAVM tissue. Sequential paraffin-embedded sections from eAVM lesion tissue and paired adjacent unaffected control tissue from three patients were stained for CD204 (M2 macrophage), and PDL-1 (immune response marker). Normal vessel is indicated by black arrows, and abnormal eAVM vessel by red arrows. Representative immunohistochemical images demonstrate that PD-L1 expression is enriched around abnormal-appearing eAVM vessels in regions identified by sequential staining as being enriched for CD204^+^ M2 macrophages, whereas normal tissue shows minimal to no PD-L1 expression or M2 macrophage infiltration. Magnification = 20x and 40x; Bar = 200 μm and 100 μm.

M2 macrophages create a locally immunosuppressive microenvironment via the secretion of PD-L1, an immune regulatory protein that suppresses immune responses by acting as a checkpoint or “brake” on immune cell activation [55,56,57,58,59]. To determine whether markers of an immunoregulatory microenvironment are present within eAVMs, we profiled the expression of PD-L1 in eAVM tissue samples compared to adjacent normal control tissues using immunohistochemistry (Figure 4). PD-L1 immunoreactivity was more prominently detected in regions enriched with CD204^+ve^ M2 macrophages, again surrounding the abnormal-appearing vessels in eAVM tissues, while PD-L1 expression was nearly undetectable or absent in both “normal” appearing eAVM BVs and all BVs in the normal control tissues (Figure 4). Together with our previous results, these data suggest an association between CD31^−ve^ vessels undergoing EndMT and localized enrichment of M2 macrophages and PD-L1 expression in eAVMs. However, additional functional studies will be required to determine whether these processes are mechanistically linked.

### 3.3. Elevation of Circulating Biomarkers of M2 Polarization and Immunosuppression in eAVM Patients’ Serum

**Figure 5:**
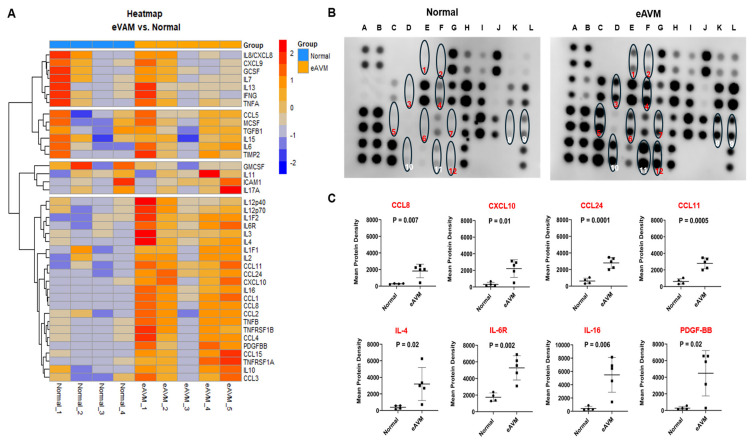
Elevated M2 immune-associated secretory factors in eAVM patient serum. (**A**) Heatmap of normalized expression (z-scores) of 40 inflammation-associated proteins measured in serum collected from five eAVM patients and four aged-matched healthy control subjects using a human inflammation antibody array. Hierarchical clustering reveals a distinct inflammatory profile in eAVMs, enriched for proteins involved in immune regulation, inflammation, and vascular remodeling. (**B**) Representative antibody array membranes from normal control and eAVM serum samples. Circled spots indicate 1. CCL11, 2. CCL24, 3. IL-4, 4. IL-6R, 5. IL-16, 6. CXCL10, 7. CCL8, 8. CCL4, 9. CCL15, 10. TNFβ, 11. TNFRSF1B, and 12. PDGF-BB proteins increased in eAVM samples. (**C**) Quantification of selected significantly upregulated proteins (text color red) in eAVM serum, including CCL8, CXCL10, CCL24, CCL11, IL-4, IL-6R, IL-16, and PDGF-BB. Data are presented as mean protein density ± SEM. Statistical significance was determined using an unpaired two-tailed *t*-test, with *p* values shown.

Currently, there are no validated circulating biomarkers that correlate with eAVM diagnosis, prognosis, or treatment response, although increased levels of several secretory proteins have been detected in the serum of patients with a variety of vascular disorders including eAVMs [60,61,62]. Because M2 polarization and PD-L1 expression were associated with a localized immune-regulatory microenvironment in eAVM tissues, we sought to determine whether circulating immune-associated proteins were differentially expressed in the serum of eAVM patients using a commercially available protein array (Figure 5A). Fifteen of the forty proteins demonstrated significantly increased mean protein density in eAVM serum compared with controls. The complete quantitative dataset for all 40 proteins analyzed in the human inflammation antibody array is provided in Table S2. Several of these proteins have established roles in immune regulation, inflammation, vascular remodeling, and macrophage-associated biological processes, although their functions are context-dependent. For example, circulating PDGF-BB (15-fold, *p* = 0.02), IL-16 (12-fold, *p* = 0.006), TNFRSF1B (9-fold, *p* = 0.02), IL-4 (8-fold, *p* = 0.02), CXCL10 (7-fold, *p* = 0.01), CCL8 (6-fold, *p* = 0.007), CCL15 (6-fold, *p* = 0.01), TNF-β (6-fold, *p* = 0.01), CCL24 (5-fold, *p* = 0.0001), and CCL4 (5-fold, *p* = 0.01) showed increased mean protein density in eAVM serum compared with controls (Figure 5B,C). Because this analysis was exploratory and performed on a limited sample size, formal multiple-comparison correction was not applied. Therefore, the differentially expressed proteins should be considered candidate immune-associated biomarkers requiring validation in larger independent cohorts.

## 4. Discussion

The presence of local inflammation and infiltration of immune cells in eAVMs has been established previously, but the role of macrophages in eAVM progression is unknown. Our findings demonstrate a selective enrichment of M2-polarized macrophages at CD31^−ve^ eAVM BVs, a locally immunosuppressive microenvironment as indicated by PD-L1 expression, and an increase in circulating indicators of immunosuppression in the serum of eAVM patients. Together with our previous observation that CD31^−ve^ regions in eAVMs are undergoing EndMT [18], these data raise the possibility that EndMT and M2 polarization are associated with pathological features of eAVMs. Given the limited number of human tissue and serum samples analyzed, these observations should be considered hypothesis-generating and require confirmation in larger patient cohorts and future mechanistic studies.

Macrophages are increasingly recognized as critical regulators of angiogenesis and vascular remodeling. While early work classified macrophages into discrete M1 (pro-inflammatory) and M2 (anti-inflammatory, pro-angiogenic) subsets, it is now clear that macrophages exist along a functional spectrum shaped by local cues [63,64]. Although the M1/M2 classification framework is widely used to describe macrophage polarization states, macrophage phenotypes in diseased tissues likely exist along a dynamic functional spectrum rather than as discrete subtypes. Recent single-cell transcriptomic studies have identified multiple macrophage populations with overlapping inflammatory, angiogenic, and immunoregulatory functions, including C1QC+ macrophage subsets associated with tissue remodeling and immune regulation. Therefore, the terminology used in the current study primarily reflects established marker-based characterization (CD163, CD204, HLA-DR) of macrophage-enriched populations rather than definitive macrophage lineage states [64,65]. We also acknowledge that HLA-DR is not a specific marker of M1 macrophages and may be expressed by multiple antigen-presenting immune cell populations, including dendritic cells, activated monocytes, and B cells. Therefore, HLA-DR staining in the present study should be interpreted as a marker-based characterization of macrophage-associated immune populations rather than definitive identification of M1 macrophages. Future studies incorporating additional canonical M1 markers, such as CD80, CD86, and iNOS, together with broader immune-cell profiling, will provide a more comprehensive characterization of macrophage phenotypes within the eAVM microenvironment. Among these populations, M2-like macrophages have been associated with the secretion of growth factors such as VEGF and PDGF, matrix-remodeling enzymes, and immune-regulatory molecules that support aberrant angiogenesis and tissue expansion [66,67]. Our observation that M2 macrophages cluster around abnormal CD31^−ve^ vessels suggests an association between macrophage enrichment and pathological vascular remodeling in eAVMs.

Importantly, our findings parallel emerging data from bAVMs, where macrophage infiltration has been associated with lesion growth, rupture risk, and hemorrhage. Prior studies have shown that macrophages are the dominant immune cell population in bAVMs, even in unruptured lesions, and that higher macrophage burden correlates with lesion severity [27,28,31,34]. M2-polarized macrophages are particularly enriched in ruptured bAVMs and are thought to promote vascular fragility through excessive angiogenic and matrix-degrading activity [28,68,69,70,71]. Our data extend these observations to eAVMs and suggest that shared inflammatory mechanisms may underlie both cranial and extracranial disease.

The selective association of M2 macrophages with abnormal CD31^−ve^ vessels is especially noteworthy. Loss of CD31 expression may reflect endothelial dysfunction, altered endothelial identity, or vascular remodeling associated with EndMT rather than simple endothelial loss. CD31 is an important regulator of endothelial integrity, vascular stability, and immune cell interactions, and reduced expression may identify regions undergoing endothelial plasticity and pathological vascular remodeling [18,19]. We have previously demonstrated that eAVMs contain a heterogeneous vascular population, including vessels with altered endothelial identity and disrupted mural cell coverage [18]. We also showed that CD31^−ve^ regions in eAVMs undergo EndMT, suggesting that endothelial plasticity may contribute to the abnormal vascular phenotype observed in these lesions. EndMT has been increasingly recognized as an important mechanism in vascular remodeling and immune modulation [13,43]. ECs undergoing mesenchymal transition can acquire altered transcriptional and cytokine profiles that favor recruitment of inflammatory cells and polarization of macrophages toward an M2 phenotype [14,72,73]. Conversely, M2 macrophages secrete mediators such as TGF-β, PDGF, and IL-10 that are known to promote mesenchymal transition and extracellular matrix remodeling [15,74,75]. These factors may further destabilize abnormal vessels and reinforce a permissive angiogenic microenvironment. Together, these observations raise the possibility of a bidirectional interaction between EndMT-associated endothelial changes and M2 macrophage polarization. Such reciprocal signaling may establish a feed-forward loop in which abnormal vessels favor macrophage recruitment and polarization, while M2 macrophages further influence vascular instability and lesion progression in eAVMs. However, because our study is observational and descriptive, these interactions should currently be considered hypothesis-generating rather than mechanistically demonstrated. Similar reciprocal interactions between abnormal endothelium and macrophages have been described in tumors and other vascular pathologies [16,74,76].

The increased expression of PD-L1 within eAVM tissues is consistent with a potentially immunoregulatory microenvironment. PD-L1 is widely expressed by M2 macrophages and functions as a key immune checkpoint that dampens local immune responses [77,78,79,80]. In cancer, PD-L1-expressing macrophages contribute to immune evasion and sustained tissue growth [81,82], and similar mechanisms may operate in eAVMs. By suppressing immune surveillance and resolution pathways, PD-L1-positive macrophages may be associated with conditions that favor persistence of abnormal vessels. This raises the intriguing possibility that immune checkpoint pathways, traditionally studied in oncology, may also be relevant therapeutic targets in vascular malformations.

Our serum cytokine profiling further supports the presence of a systemic immunosuppressive milieu in eAVM patients. The marked elevation of M2-associated factors, including PDGF-BB, IL-4, IL-16, and multiple chemokines, suggests that macrophage polarization in eAVMs is not confined to the lesion itself but may reflect broader immune dysregulation. PDGF-BB, in particular, is a potent regulator of mural cell recruitment and vascular remodeling, and its elevation may be associated with both immune signaling and abnormal vessel architecture observed in eAVMs [83,84]. These circulating factors could also serve as potential biomarkers of disease activity or progression. These findings may also have therapeutic implications. In bAVMs, macrophage infiltration and inflammatory signaling have been associated with vascular instability, lesion severity, and hemorrhage risk, suggesting that immune-mediated vascular remodeling may be relevant across arteriovenous malformation subtypes [27,29]. In tumor models, PD-L1-expressing macrophages and M2-like macrophage polarization have been linked to immune regulation and tissue remodeling, and therapeutic strategies targeting PD-1/PD-L1 pathways or macrophage repolarization are under active investigation [57,58,81,85,86]. Although such approaches have not yet been established for eAVMs, our findings raise the possibility that macrophage polarization and immune-regulatory signaling may represent future therapeutic targets. In parallel, genotype-directed therapies for eAVMs, including MEK inhibition with trametinib in MAP2K1-mutant lesions, support the broader concept that targeted modulation of vascular signaling pathways may improve treatment options for aggressive vascular malformations [12,87].

This study has several limitations. First, the analyses are primarily descriptive and based on spatial associations observed in human tissue specimens. Although our findings demonstrate enrichment of M2 macrophages around abnormal CD31^−ve^ vessels, they do not establish direct mechanistic causality between macrophage polarization and lesion progression. Because this study is based on observational analyses of human tissue and serum samples, causal relationships between EndMT, macrophage polarization, PD-L1 expression, and disease progression cannot be established. Second, macrophage characterization in the present study was based on a limited panel of established markers. Although CD163 and CD204 are widely used markers of M2-like macrophages, HLA-DR is not specific for M1 macrophages and may also be expressed by other antigen-presenting immune cell populations. Therefore, the macrophage populations identified in this study should be interpreted as marker-associated populations rather than definitive macrophage phenotypes. Future studies incorporating additional canonical M1 markers, such as CD80, CD86, and iNOS, together with broader immune-cell profiling, will further refine characterization of the eAVM immune microenvironment. Third, the sample size was limited due to the rarity of human pediatric eAVMs. In addition, the tissue immunostaining findings are qualitative and based on representative images from a limited number of human specimens. Quantitative image analysis was beyond the scope of the present study and should be incorporated into future investigations. Finally, functional studies evaluating reciprocal signaling between EndMT-associated endothelial cells, macrophages, and PD-L1 were beyond the scope of the current study. Future studies using quantitative spatial analysis, co-culture systems, and mechanistic signaling approaches will be important to further define these interactions.

In conclusion, our study identifies M2 macrophage enrichment and immune regulatory features associated with the eAVM microenvironment. Because the present study is exploratory and observational in nature, these findings should be interpreted as generating testable hypotheses regarding the relationship between EndMT-associated vascular remodeling, macrophage polarization, and immune regulation in eAVMs rather than establishing causal mechanisms. By demonstrating associations between abnormal vessel structure and localized and systemic immune dysregulation, these findings shift the conceptual framework of eAVMs from purely developmental vascular defects to dynamic, inflammation-driven lesions. This work lays the foundation for future studies aimed at modulating macrophage behavior or immune signaling as a novel therapeutic strategy for eAVMs and potentially other aggressive vascular malformations.

## Data Availability

The original contributions presented in this study are included in the article/Supplementary Material. Further inquiries can be directed to the corresponding author.

## Supplementary Materials

The following supporting information can be downloaded at https://www.mdpi.com/article/10.3390/biomedicines14071519/s1. Figure S1: M2 macrophages are enriched around abnormal vessels in eAVM tissue. Sequential paraffin-embedded eAVM lesion tissue and paired adjacent unaffected control tissue sections from three patients were stained for CD163 (M2 macrophage), HLA-DR (M1 macrophage), and CD31 (mature EC marker). Normal-caliber CD31^+ve^ vessels are indicated by black arrows and dilated CD31^−ve^ vessels by red arrows. CD163^+ve^ M2 macrophages preferentially localize around CD31^−ve^ abnormal vessels, whereas HLA-DR^+ve^ M1 macrophages are associated with normal CD31^+ve^ vessels and normal tissue. Magnification = 20x and 40x; Bar = 200 μm and 100 μm. Figure S2. Representative immunofluorescence images showing expression of CD31 and CD163 in eAVM lesion tissue compared with paired adjacent unaffected control tissue: Abnormal CD31^−ve^ eAVM vessels express CD163, whereas adjacent CD31^+ve^ normal vessels do not express CD163. Magnification =20x and 40x; Bar = 200 μm and 100 μm. Table S1. Antibodies used for immunohistochemistry and immunofluorescence. Table S2: Complete quantitative data for the 40 inflammation-associated proteins analyzed using the the human inflammation antibody array.

## Abbreviations

eAVMs	Extracranial arteriovenous malformations
IHC	Immunohistochemistry
IF	Immunofluorescence
SNVs	Single-nucleotide variants
EC	Endothelial cell
EndMT	Endothelial-to-mesenchymal transition
MCs	Mural cells
bAVMs	Brain arteriovenous malformations
TAMs	Tumor-associated macrophages
