# Supplementary material for: M2 Macrophage Polarization Characterizes an Immunosuppressive Microenvironment in Extracranial Arteriovenous Malformations
Source: Biomedicines. 2026 Jul 7;14(7):1519. doi: 10.3390/biomedicines14071519 (PMC13406079; doi:10.3390/biomedicines14071519)
Supplement: Supplementary file 1 [file biomedicines-14-01519-s001.zip › biomedicines-4400847-supplementary.pdf]

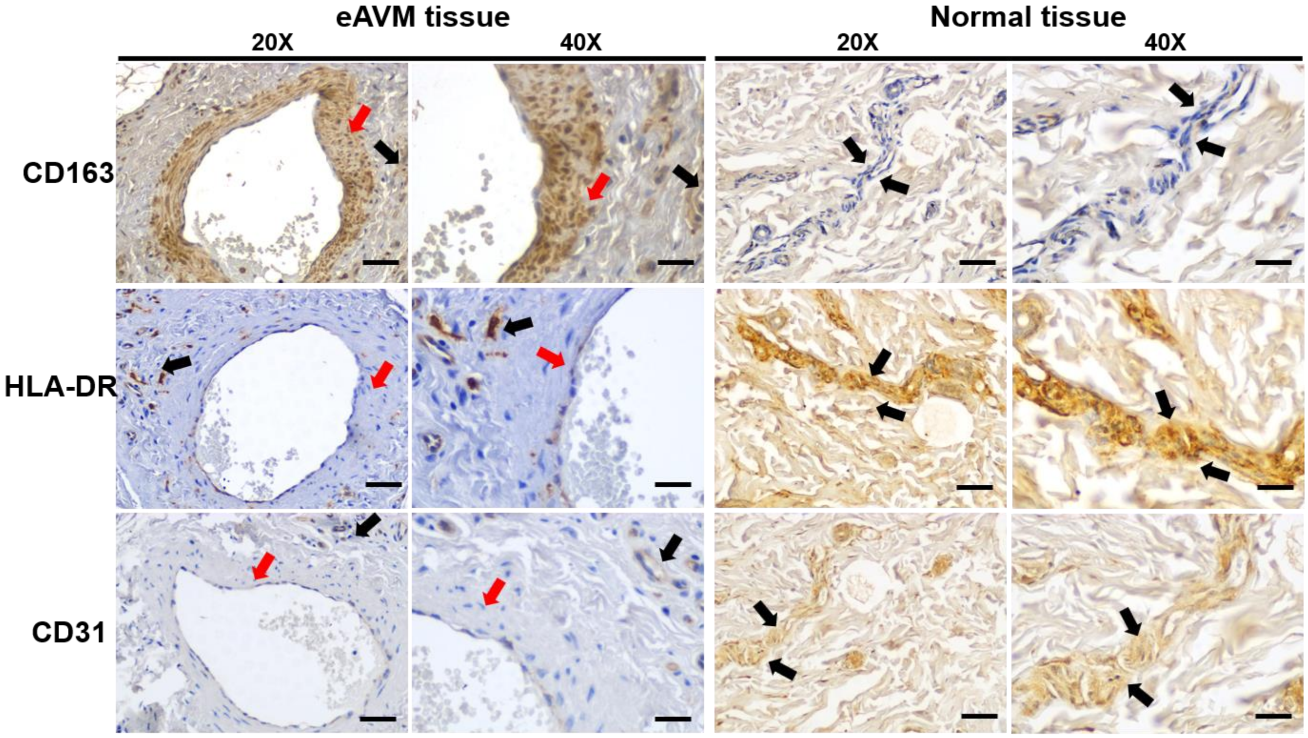


**Figure S1:** M2 macrophages are enriched around abnormal vessels in eAVM tissue. Sequential paraffin embedded eAVM lesion tissue and paired adjacent unaffected control tissue sections from three patients were stained for CD163 (M2 macrophage), HLA-DR (M1 macrophage), and CD31 (mature EC marker). Normal-caliber $CD31^{+ve}$ vessels are indicated by black arrows, and dilated $CD31^{-ve}$ vessels by red arrows. $CD163^{+ve}$ M2 macrophages preferentially localize around $CD31^{-ve}$ abnormal vessels, whereas HLA-$DR^{+ve}$ M1 macrophages are associated with normal $CD31^{+ve}$ vessels and normal tissue. Magnification = 20x and 40x; Bar = 200 μm and 100 μm.

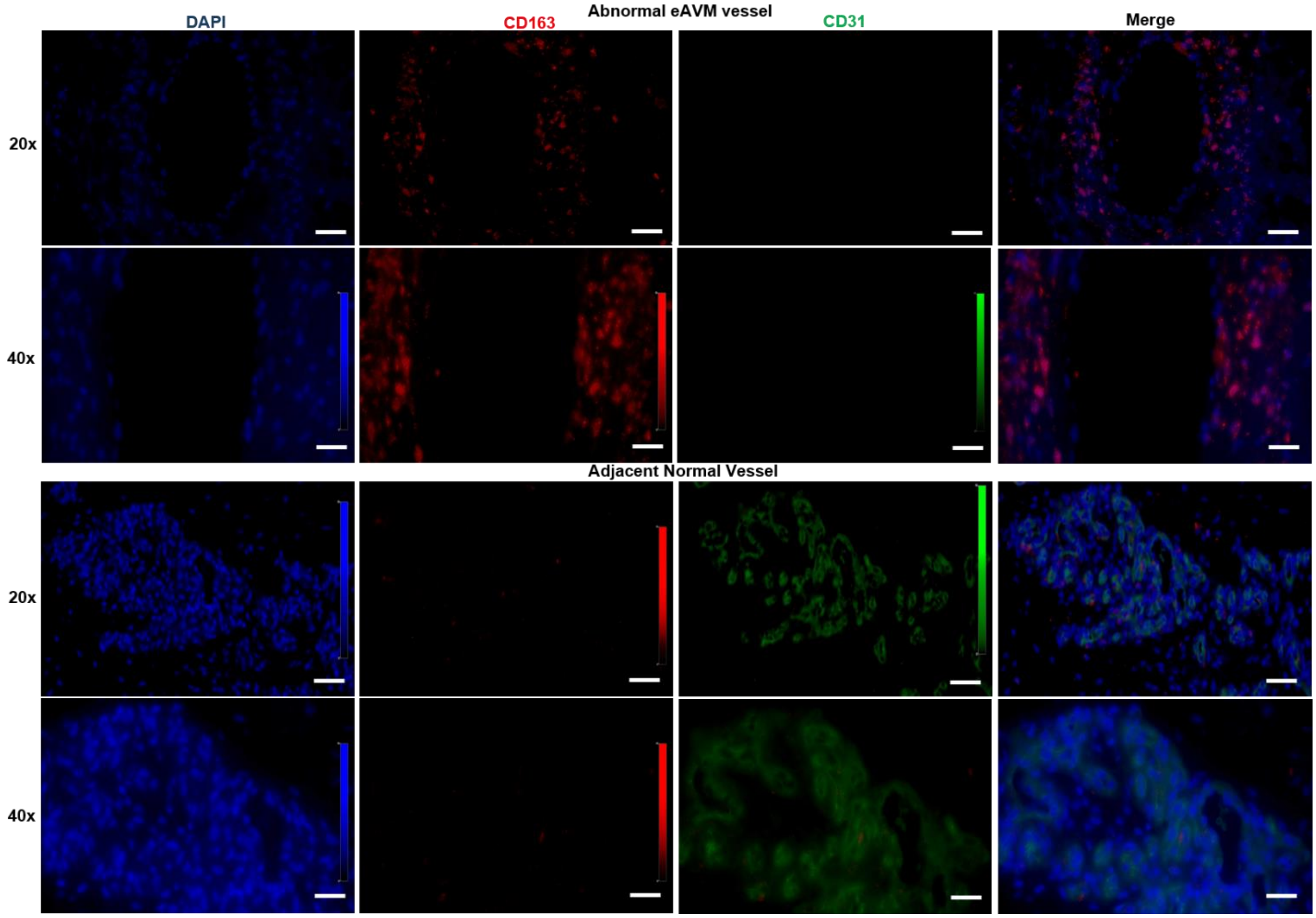


**Figure S2.** Representative immunofluorescence images showing expression of CD31 and CD163 in eAVM lesion tissue compared with paired adjacent unaffected control tissue: Abnormal CD31$^{-ve}$ eAVM vessels express CD163, whereas adjacent CD31$^{+ve}$ normal vessels do not express CD163 . Magnification =20x and 40x; Bar = 200 µm and 100 µm.

**Table S1. Antibodies used for Immunohistochemistry, and immunofluorescence.**

| Protein | Application | Usage | Cat. No | Company | Host | Used in |
|---|---|---|---|---|---|---|
| CD31 | IHC, IF | 1:800 | 3528s | Cell Signaling | Mouse | IHC; IF with CD163 |
| CD163 | IHC | 1:50 | PA5-109327 | Invitrogen | Rabbit | IHC |
| CD163 | IHC, IF | 1:100 | TA506391 | Invitrogen | Mouse | IF with CD204 |
| CD163 | IF | 1:1000 | MA5-54106 | Invitrogen | Rabbit | IF with CD31 |
| CD204 | IHC, IF | 1:100 (IHC) 1:50 (IF) | Ab271070 | Abcam | Rabbit | IHC; IF with CD163 |
| HLA-DR | IHC | 1:100 | A500-022A | Fortis | Mouse | IHC |
| PDL-1 | IHC | 1:5000 | 66248-1-1g | Proteintech | Mouse | IHC |
| Goat anti-Mouse IgG (H+L) Secondary Antibody, FITC | IF | 1:100 | 31569 | Invitrogen | | CD31 in CD31/CD163 CO-IF |
| Goat anti-Rabbit IgG (H+L) Secondary Antibody, TRITC | IF | 1:100 | 31670 | Invitrogen | | CD163 in CD31/CD163 CO-IF |
| Alexa Fluor™ 594 Goat anti-Mouse IgG | IF | 1:100 | A-11032 | Invitrogen | | CD163 in CD163/CD204 CO-IF |
| Alexa Fluor™ 488 Goat anti-Rabbit IgG | IF | 1:500 | A-11008 | Invitrogen | | CD204 in CD163/CD204 CO-IF |

**Table S2: Complete quantitative data for the 40 inflammation-associated proteins analyzed by the human inflammation antibody array.**

| S. No. | Protein Name | Mean Protein Density (Normal) | Mean Protein Density (eAVM) | Fold Change | P-value |
|---|---|---|---|---|---|
| 1 | IL-8/CXCL8 | 5087.3965 | 4272.4686 | 0.84 | 0.689 |
| 2 | CXCL9 | 4622.1245 | 4154.3344 | 0.90 | 0.870 |
| 3 | GCSF | 4241.575125 | 3571.0088 | 0.84 | 0.718 |
| 4 | IL-7 | 2530.86625 | 1931.0936 | 0.76 | 0.676 |
| 5 | IL-13 | 3537.19325 | 3986.6327 | 1.13 | 0.842 |
| 6 | IFN-γ | 5075.96275 | 4620.1296 | 0.91 | 0.853 |
| 7 | TNF-α | 4480.95125 | 4786.9664 | 1.07 | 0.915 |
| 8 | CCL5 | 5779.695 | 7665.5276 | 1.33 | 0.361 |
| 9 | M-CSF | 1532.1115 | 1945.7318 | 1.27 | 0.674 |
| 10 | TGF-β1 | 5329.538 | 5815.4049 | 1.09 | 0.862 |
| 11 | IL-15 | 4954.6305 | 5040.0791 | 1.02 | 0.963 |
| 12 | IL-6 | 4129.1815 | 6008.3258 | 1.50 | 0.362 |
| 13 | TIMP-2 | 1989.819 | 3361.3091 | 1.69 | 0.404 |
| 14 | GM-CSF | 2119.82375 | 986.902 | 0.47 | 0.102 |
| 15 | IL-11 | 189.754 | 257.8516 | 1.36 | 0.637 |
| 16 | ICAM-1 | 1208.75525 | 1273.5846 | 1.05 | 0.896 |
| 17 | IL-17A | 214.404 | 325.0706 | 1.52 | 0.655 |
| 18 | IL-12p40 | 1526.3355 | 4430.7806 | 2.90 | 0.134 |
| 19 | IL-12p70 | 3031.63275 | 7737.801 | 2.55 | 0.074 |
| 20 | IL-1F2 | 638.23675 | 2928.7442 | 4.59 | 0.004 |
| 21 | IL-6R | 1745.42625 | 5275.6314 | 3.02 | 0.002 |
| 22 | IL-3 | 1328.77925 | 4739.2297 | 3.57 | 0.080 |
| 23 | IL-4 | 390.41425 | 3197.8524 | 8.19 | 0.027 |
| 24 | IL-1F1 | 469.9255 | 829.709 | 1.77 | 0.206 |
| 25 | IL-2 | 440.5635 | 1011.0754 | 2.29 | 0.057 |
| 26 | CCL11 | 618.3735 | 2794.7159 | 4.52 | 0.0005 |
| 27 | CCL24 | 293.178 | 1730.9394 | 5.90 | 0.0001 |
| 28 | CXCL10 | 319.2815 | 2216.7445 | 6.94 | 0.010 |
| 29 | IL-16 | 433.02275 | 5490.1238 | 12.68 | 0.006 |
| 30 | CCL1 | 312.7395 | 851.763 | 2.72 | 0.002 |
| 31 | CCL8 | 282.139 | 1826.6077 | 6.47 | 0.007 |
| 32 | CCL2 | 715.50275 | 1767.0673 | 2.47 | 0.061 |
| 33 | TNF-β | 641.13275 | 4264.1694 | 6.65 | 0.017 |

| S. No. | Protein Name | Mean Protein Density (Normal) | Mean Protein Density (eAVM) | Fold Change | P-value |
|---|---|---|---|---|---|
| 34 | TNFRSF1B | 659.399 | 6030.3164 | 9.15 | 0.022 |
| 35 | CCL4 | 724.39525 | 4183.435 | 5.78 | 0.017 |
| 36 | PDGF-BB | 284.569 | 4484.798 | 15.76 | 0.026 |
| 37 | CCL15 | 759.91525 | 4531.3428 | 5.96 | 0.016 |
| 38 | TNFRSF1A | 377.709 | 1677.3 | 4.44 | 0.045 |
| 39 | IL-10 | 1662.21775 | 4358.7638 | 2.62 | 0.100 |
| 40 | CCL3 | 1857.69775 | 5339.7188 | 2.87 | 0.065 |

**Note:** Values represent mean protein density obtained from the human inflammation antibody array. Fold change was calculated as mean protein density in eAVM divided by mean protein density in normal controls. Statistical significance was determined using an unpaired two-tailed Student's t-test.
